# Association between Dining with Companions and Depression among Korean Adults

**DOI:** 10.3390/nu14142834

**Published:** 2022-07-10

**Authors:** Hankyo Jung, Jin Ryu, Junhui Choi, Hyunkyu Kim, Euncheol Park

**Affiliations:** 1 Premedical Courses, Yonsei University College of Medicine, Seoul 03722, Korea; lawofgravity@yonsei.ac.kr (H.J.); ryujin1905@naver.com (J.R.); ch0629jh@naver.com (J.C.); 2 Department of Preventive Medicine, Yonsei University College of Medicine, Seoul 03722, Korea; 3 Institute of Health Services Research, Yonsei University, Seoul 03722, Korea; 4 Department of Psychiatry, Yonsei University College of Medicine, Seoul 03722, Korea

**Keywords:** depression, dining with companions, meal companionship, meal frequency

## Abstract

We investigated whether dining with companions is correlated with the alleviation of depression and differs by sex among Korean adults. We used 4-year data from the 2014, 2016, 2018, and 2020 Korea National Health and Nutrition Examination Survey. We surveyed 11,055 participants (4699 men, 6356 women) using the Patient Health Questionnaire-9 to measure their depression scores. We evaluated participants’ meal companionship status by asking whether they had usually dined with companions for breakfast, lunch, or dinner during the past year. Statistical analysis was performed using chi-square tests and multivariate/multinomial logistic regression. We found depression to be less prevalent among participants who dined with companions at least once a day (adjusted OR: 0.26, 95% CI: 0.15–0.45, men; adjusted OR: 0.50, 95% CI: 0.34–0.74, women). In the moderate depression subgroup, participants who dined with companions at least once a day showed lower OR (adjusted OR: 0.28, 95% CI: 0.16–0.50, men; adjusted OR: 0.50, 95% CI: 0.32–0.76, women). Among participants who dined together, men’s severe depression dramatically decreased (adjusted OR: 0.05, 95% CI: 0.01–0.31). Thus, we found an association between dining with companions and the prevalence of depression among Korean adults. Dining with companions compared with dining alone signified a lower depression rate, especially among men. This study can provide an initiative to further analyze psychological and physiological effects of dining together and be applied to practical fields as education and societal campaigns.

## 1. Introduction

Depression is still considered one of the most common and prominent mental illnesses. Rates of depression have continued to rise; for example, the prevalence of major depressive disorders (MDD) increased between 2000 and 2010 in France [1]. The prevalence of depression in the USA also increased significantly from 2005–2015, encouraging mental health intervention efforts [2]. An epidemiological study warned that depression would be more common in South Korea, as MDD rates in Korean adults ranged from 3.3–5.6% in 2011 [3]. More recently, the COVID-19 pandemic has negatively impacted people around the world by causing anxiety and depression [4,5,6]. Individuals’ mental state is being threatened by COVID-19 in terms not only of one’s unstable household income but also of increased depression [7,8]. This affirms the idea that depression is now a worldwide problem for which proper solutions should be offered.

Depression requires pre-emptive diagnosis and treatment because it can lead to personal and social tragedies such as suicide. In fact, suicide attempts are readily made by patients with depression or other psychiatric disorders [9,10,11]. Considering the possible economic burden and clinical complications that accompany depression, the need for predictors for depressive symptoms is increasing [12,13,14].

People with depression are inclined to be less responsive to social interactions, finding it less enjoyable to interact with others on a daily basis [15,16]. However, the positive effect of social interactions still holds for individuals with mental illnesses. Social ties can act as a catalyst for psychological well-being and recovery from depression [17]. However, according to the Korean Statistical Information Service (KOSIS), the number of single-person households in Korea has been rapidly increasing (31.7% in 2020). This raises the possibility that time spent with other people outside of one’s workplace may decrease. One marker can be meal companionship, and prior research in Western society highlights the decreased frequency of dining with others when living alone [18]. The need to interact with companions by dining with them provides a tool to help monitor depression signs.

Several studies have established that dining alone is far from beneficial for mental health. In an attempt to measure health-related quality of life among Korean adults, a lower frequency of dining together was associated with a lower EuroQol five-dimensional index and more problems [19]. A cross-sectional study of Korean adults showed that people who had dinner alone indicated 1.308 and 1.531 times higher rate of stress and depression, respectively, compared with those who had dinner with others [20]. These findings suggest that the inclination of dining alone is associated with lower mental health and well-being, which has not yet been confirmed for people with varied demographic features using another tool for screening depression. However, the objective of this paper is not limited to re-verifying a possible correlation between variables; we desired to focus on finding more elaborate indicators of the relationship with depression. Such attributes would encompass differences by sex, degrees of depression, and forms of dining frequency.

Although “eating” is an important part of one’s life, there are few in-depth studies on the relationship between dining together and mental health in Korea. Most studies focus on older adults, do not discuss meal companions other than family, or only target a certain meal type such as dinner [21,22,23]. Moreover, more prudent approaches by adjusting for confounding variables including skipping meals need to be deployed.

Accordingly, this study aimed to investigate whether dining with others is correlated with the alleviation of depression among Korean adults. The correlation was investigated separately by sex and further analyzed for different levels of depression and dietary patterns.

## 2. Methods

### 2.1. Study Population and Data

The data used in this study were obtained from the results of the 2014, 2016, 2018, and 2020 Korea National Health and Nutrition Examination Survey (KNHANES). Four-year data were combined to ensure stable statistical analysis based on sufficient samples. The integrated weight value of each year data was calculated by simple averaging, according to the KNHANES Instructions on Raw Data Analysis for SAS software (version 9.4, SAS Institute, Cary, NC, USA). KNHANES is an annual nationwide survey in South Korea performed by the Korea Disease Control and Prevention Agency (KDCA). The official goal of KNHANES is to acquire statistical information on the health status, health-related consciousness and behaviors, and food consumption and nourishment status of Korean citizens. Since the Patient Health Questionnaire-9 (PHQ-9) as a selective survey tool for identifying depressive adults was only included in every even year, the data from the corresponding years from 2014–2020 were included in this research. The study population exclusively comprised Korean adults (age ≥ 19). KNHANES has been examined and approved by the Institutional Review Boards of the KDCA, which is administrated under government regulations as well as the Declaration of Helsinki. Therefore, this study conforms with ethical standards for research in medicine.

Not all of the data of Korean adults in 2014, 2016, 2018, and 2020 KNHANES were included for the study participants. People who did not have investigated data on covariates of our interests were first filtered out of the study. Then people who satisfied the condition of having breakfast, lunch, and dinner at least three times a week, respectively, were included in our final study.

### 2.2. Measures

#### 2.2.1. Patient Health Questionnaire-9 

The Patient Health Questionnaire-9 (PHQ-9) was utilized to identify depressive symptoms among participants. The PHQ-9, targeted at adults, was developed to conveniently but efficiently diagnose depression as a self-report version of the Primary Care Evaluation of Medical Disorders [24]. Based on DSM-IV diagnosis criteria, the PHQ-9 questionnaire consists of 9 items regarding problematic symptoms over the last two weeks, each of which is scored on a 4-point rating scale ranging from 0 (not at all) to 3 (nearly every day) [25]. With the total score summed up to 27, the cutoff level of 10 out of 27 or higher was validated as yielding a sensitivity and specificity rate of 88% for major depression [25]. Applying the identical threshold for depression as a total score of 10, the Korean version of the PHQ-9 test reported the AUROC (area under the receiver operating characteristic curve) to be an excellent value of 0.944 (*p* < 0.05) [26]. Another Korean-version study with a cutoff level of 10 showed high values of sensitivity and specificity, 87.8% and 97.4%, respectively [27]. In this study, we defined participants as “depressive” when the total PHQ-9 score was 10 or higher. In addition, sensitivity analyses were performed with cutoff scores of 5, 10, and 20 as dissimilar depression subgroups to check the robustness of our statistics model.

#### 2.2.2. Meal Companionship Related Variables

To inspect meal companionship status, study participants were asked a question about whether they usually had breakfast with companions over the last year. People whose breakfast frequency per week was less than three times were excluded from research observations regardless of their responses, considering insufficiency of breakfast frequency per se. People who replied “yes” were asked to answer the subsequent question regarding who they usually had breakfast with. Using the same format, questions and the exclusion method above were repeated for other meals, namely, lunch and dinner.

According to the meal type, participants could answer “no” or “yes” to each independent question about their meal companionship. Counting “no” as 0 and “yes” as 1, participants would therefore have a frequency spectrum for dining together ranging from 0–3 times per day. Next, people who “did not” dine with companions (0 time per day) were classified into the group “meals alone”, whereas those who “did” dine with companions (1~3 times per day) were added to the group “meals together”.

#### 2.2.3. Covariates

The demographics included age, educational attainment, equalized household income, marital status, and residential area for socioeconomic covariates. Smoking status, alcohol use status, and BMI were included to display the health-related characteristics of the participants. The number of household members was added to take one’s usual household environment into account. Physical activity, divided into three groups by metabolic equivalent task, according to the guidelines for data processing and analysis of the International Physical Activity Questionnaire (IPAQ) published by the IPAQ Research Committee, was also added to covariates.

### 2.3. Statistical Analysis

Chi-square tests were performed to examine and compare statistical correlations between each independent variable and depression. Multivariate logistic regression analysis was applied to verify the association between meal companionship and depression, with other demographical variables included in the analysis. Subgroup analyses were conducted using multivariate logistic regression analyses between investigated variables and depression, along with multinomial logistic regression analyses between meal companionship and depression after categorizing depression into more detailed levels. All statistical analyses were performed using SAS software, version 9.4 (SAS Institute, Cary, NC, USA). Results are presented with adjusted odds ratios (ORs) and 95% confidence intervals (CIs). A *p*-value < 0.05 was set as the condition to indicate statistical significance of the results.

## 3. Results

### 3.1. Participants’ General Characteristics

Table 1 shows the overall characteristics of the participants across the listed variables. A total of 11,055 participants (4699 men, 6356 women) were the target population. The mean and standard deviation values for age were 54.5 ± 16.5 (men), 53.6 ± 15.5 (women), and 54.0 ± 16.0 (total). The mean and standard deviation values for BMI were 24.4 ± 3.2 (men), 23.5 ± 3.4 (women), and 23.9 ± 3.4 (total).

PHQ, being the sum of scores of the PHQ-9 test, 2.8% of men and 5.3% of women reported PHQ ≥ 10 which indicated being “depressive.” The prevalence of depression was less among those who dined with companions at least once a day than in people who had meals alone. Compared with the rate of depression in “meals together”, the percentage rate of depression in “meals alone” was approximately 5.4 and 2.8 times higher in men and women, respectively. Whether or not people usually dined with companions was statistically significant for both men and women, as were variables including educational attainment, equalized household income, marital status, smoking status, and the number of household members. Regarding the prevalence of depression, BMI was statistically significant only in men, whereas age and year variables were significant only in women.

### 3.2. Association between Investigated Variables and Depression

The results of the multivariate logistic regression between meal companionship and depression are shown in Table 2. Taking people who dined alone as a reference group, those who usually dined with companions were less likely to become depressive. In particular, men (adjusted OR: 0.26, 95% CI: 0.15–0.45) showed stronger association between dining together and decreased depression than women (adjusted OR: 0.50, 95% CI: 0.34–0.74). For both men and women, a consistent tendency was found for equalized household income and smoking status, that is, equalized household income was inversely associated with depression. Regardless of gender, smokers showed twice the likelihood or more of depression than non-smokers.

Men in their 30s were about three times more depressive than those aged 19–29. Women over 70 were about three times less depressive than those aged 19–29. None of the other age groups showed any statistical association with depression. Unlike men where educational attainment was not certainly correlated with depression, women with higher educational attainment tended to be less depressive. Marital status of being separated/divorced/widowed was related to a higher depression rate in women.

Most of the other variables, such as alcohol use status, residential area, BMI, number of household members, and physical activity had no statistically significant relationship with depression. 

### 3.3. Subgroup Analyses by Covariates

Table 3 shows the results of multivariate logistic regression to examine the correlation between dining together within each covariate subgroup and depression. Among men, slightly more subgroups showed a statistical association with decreased depression when dining together than among women. Among subgroups in which both men and women yielded statistically significant CIs, all of the adjusted OR values of depression were smaller in men.

Heterogeneity was found in a series of years in that the adjusted OR values of dining together greatly vary by years. Years when significant values appear were also opposite by gender. This inconsistency in time subgroups could reasonably occur, owing to the rolling sample method embedded in KNHANES data processing [28]. Data were obtained from separated periods of time; 2013–2015 (sixth survey), 2016–2018 (seventh survey), 2019–2020 (eighth survey), which accounts for the diversity in the statistics. It can also be interpreted that whether individuals dine with someone else is not seriously affected by year, at least during the given four years.

### 3.4. Subgroup Analyses by Levels of Depression

Total PHQ-9 scores of 5, 10, 15, and 20 have been suggested to demarcate the lower limits of mild, moderate, moderately severe, and severe depression, respectively [25]. Our study adopted the revised thresholds of 5, 10, and 20 for mild, moderate, and severe depression, respectively, which was proposed in the 2020 Standardized Instructions on Mental Health Screening Methodologies, published by the National Center for Mental Health (http://www.ncmh.go.kr/, accessed on 17 May 2022).

**Figure 1:**
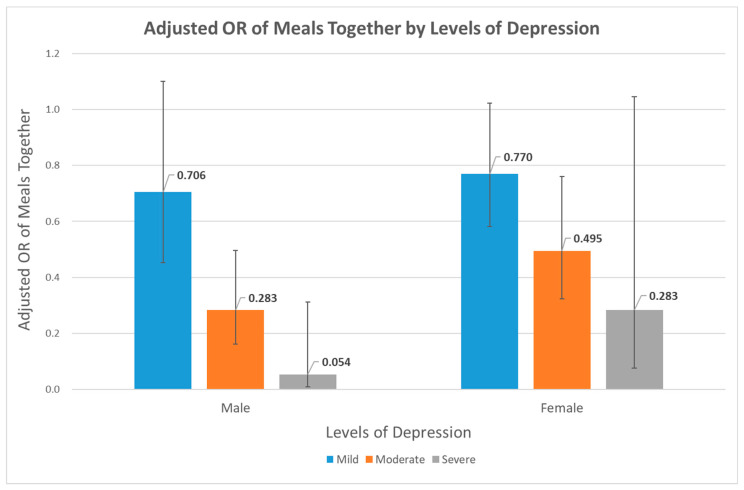
Multinomial logistic regression analysis for the association between having meals together and detailed levels of depression. Blue, orange, and grey colors each represent mild, moderate, and severe depression levels. Adjusted OR values are indicated above each bar, and black bars stand for 95% CIs.

Figure 1 depicts adjusted ORs and 95% CIs as a result of multinomial logistic regression between meal companionship and levels of depression. The predictive effects of other covariates were considered in the regression analysis. Statistical associations were found in moderate depression for both sexes (adjusted OR: 0.28, 95% CI: 0.16–0.50, men; adjusted OR: 0.50, 95% CI: 0.32–0.76, women) and in severe depression for men (adjusted OR: 0.05, 95% CI: 0.01–0.31). For both men and women with levels of worsening depression, adjusted OR values of depression when dining together declined. The numbers and percentages of study participants of each depression group are shown in Table S1.

### 3.5. Subgroup Analyses by Various Meal Patterns

**Figure 2:**
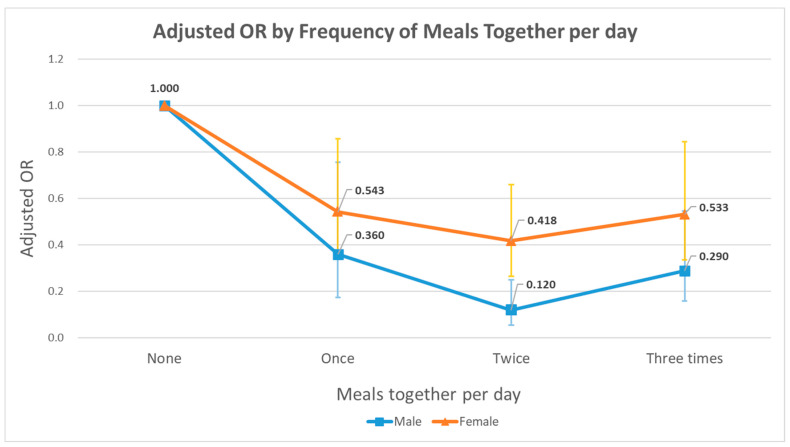
Multivariate logistic regression analysis for the association between frequency of meals together per day and depression. Blue graph and light-blue bars stand for adjusted OR values and 95% CIs of men. Orange graph and yellow bars stand for adjusted OR values and 95% CIs of women. Adjusted OR values are indicated above each point of graphs.

Additional subgroup analyses were performed to see differences by types of meals, types of companions, and frequency of dining together per day. The results derived from multivariate logistic regression considering covariates within a model are displayed in Table 4 and Figure 2. For breakfast, lunch, and dinner (types of meals), participants who dined with somebody were further asked who they dined with (types of companions). Subgroups of lunch with people other than family and dinner with family showed statistical significance in lowered depression rates.

Meanwhile, the frequency of dining together per day was categorized in detail by the number of meals together per day. The results, depicted in Figure 2, indicated similar tendencies among men and women; the adjusted OR value was lowest at the point where meals together per day was equal to twice a day.

Table S2 shows the numbers and percentages of study participants by types of meals and companions. Table S3 shows the results of multivariate logistic regression between other related meal-pattern variables and depression.

## 4. Discussion

This study has verified the association between meal companionship and reduction in the occurrence of depression. Both men and women who regularly had meals with their companions over the past year showed a significant decrease in the prevalence of depression compared with those who dined alone. Furthermore, dining together led to a larger decrease in the adjusted OR value in men than in women.

Interestingly, in a similar context, subgroup analysis by levels of depression showed a larger drop in the prevalence of moderate depression in men than in women. Men who had dined with companions further showed a greatly lowered prevalence of severe depression, whereas statistical significance was not ensured among women with severe depression. This phenomenon, the stronger association with meal companionship in men, was consistently found in this study. Another study revealed that relative to those who had all three meals together, men and women who had every meal alone were 1.72 and 1.58 times more likely to experience depression, respectively [29]. However, further research is required to confirm whether this observation can be accepted as a generalized idea or rejected as mere coincidence and to explain sex differences. In fact, as well-documented in previous studies, higher prevalence of major depression was mostly found among women than men within the general population [30,31,32,33]. This evokes the idea that the default occurrence rate of depression is higher in women, leading to a seemingly less alleviation effect of dining together in women. In the social view, the algorithms for coping with mental stress in men and women might be different [17,34]. Nonetheless, speculation grounded in sufficient study results should be warranted to see if such stress theories can be extended to depression associated with dining alone.

Table 4 reveals that having breakfast with somebody, whether family or not, did not necessarily result in the lower prevalence of depression. However, lunch with people other than family and dinner with family were particularly associated with a decrease in the rate of depression. This suggests the need for a useful policy for Korean adults to avoid depression. Company for meals, depending on the type of meal, has not been a major topic in the discussion on depression prevention. Although with whom as well as when to dine relies on one’s preferences, this can be partially explained by “the law of inertia”. According to a survey that examined usual meal partners of 663 adults within a community, most respondents ate alone at breakfast, alone or with co-workers at lunch, and with family members at dinner [35]. Given that people will feel more comfortable and less depressive under circumstances to which they are accustomed, people may prefer to maintain their communal eating patterns [36]. This can be likened to inertia, the propensity of people to willingly retain their habits: for instance, lunch with co-workers nearby and dinner with family. These habitual patterns reflected in the types of companions with whom people usually dine might represent one aspect of how the work-oriented society operates [35]. Thus, the type of one’s meal companion will be determined differently while at work and after leaving work; however, it should be basically aimed toward the avoidance of possible depression.

As shown in Figure 2, both men and women showed a flat U-shaped graph of adjusted OR values for depression by frequency of meals together. The graphs suggest that the tendencies in depression rates by the frequency of dining together are quite similar for both sexes. However, the bottom of the flat U-shape is easily detected; the adjusted OR value reached its minimum when people had meals together twice a day (not three times a day). This could be attributed to the so-called paradox of solitude, the state in which two opposite attributes of solitude collide. Solitude, on one hand, brings about loneliness and ultimately depression, but on the other, it serves self-enhancing functions alleviating depressive symptoms [37,38]. Given that, aloneliness is defined as a set of negative feelings that arise from not spending enough time alone, each individual would have their own point of equilibrium where loneliness and aloneliness stay in harmony [38]. Therefore, proper control and balancing between loneliness and aloneliness is essential. This study could be the first to propose that the general baseline for the two conflicting sentiments derived from solitude could be located at “twice a day” with regard to dining together. Follow-up research to inductively confirm this idea is needed.

Several studies have elucidated how dining alone is related to depressive symptoms, supporting our hypothesis that dining alone influences depression. A study viewing meal companionship as a process of socialization argued that commensality could create a sense of social support and integration for older adults [39]. Social participation is also expected to mitigate depression [17]. This line of thought subtly implies that dining with others can contribute to the treatment and even prevention of depression. The study on 4905 Korean adults in 2014 focused on searching for common traits of people with specific dietary habits [40]. It was stated that people who usually ate alone were more likely to live alone, have no spouse, and have lower income levels. Living alone, not being married, and being impoverished are known as possible factors of depression [41,42,43]. These characteristics that people who dine alone share can simultaneously influence depression.

There exists a perspective of seeing depression as a type of cognitive disorder. It infers that ineffective emotion regulation strategies and related cognitive malfunction underlie depression [44,45,46]. One’s cognitive ability is being probed in terms of its social aspect; social interactions enable social cognition [47]. Social interactions embodied in forms of conversations during mealtime with companions could thereby allow more precise understanding of one’s circumstances and instruct on how to behave. This process of social reflection and behavioral change in a cognitive perspective will improve skills for emotional control and reduce depressive symptoms. Thus, explanations for how dining with companions can relieve people from depression can be partially achieved. Moreover, in patients with Alzheimer’s disease, which is deeply associated with depression, studies have shown that cognitive function remained higher for patients with larger social networks [48,49]. Such discoveries are inspiring because the social network one has is thought to affect cognitive function and the possibilities of developing depression. Meal companionship is a good example of social networking, which is a good therapy for depression.

Another study using KNHANES data along with PHQ-9 measures for depression showed that depressive symptoms as well as suicidal ideation are also more likely to occur in adults who had dinner alone, compared with those who had dinner with others [50]. Moreover, the adjusted OR value for the prevalence of depression when having dinner alone was higher among men than women. Such results concur with those obtained from the present study. However, in this study, the association between meal companionship and depression was further investigated for breakfast and lunch in addition to dinner through subgroup analyses. Subgroups such as levels of depression by subdivision of the PHQ-9 score cutoffs were introduced anew in logistic regression, with statistically significant outputs.

This study suffers some intrinsic limitations. First, the study was performed as a cross-sectional study, and the causal relationship between depression and dining alone could not be clearly identified. In other words, the causation order remains unclear. People diagnosed with depression in advance might refuse to partake in social gatherings for meals, or, conversely, continued exposure to experiences of dining alone can cause depression. Second, other potential covariates could have been omitted in the regression model. Depression is reportedly interconnected with neurodegenerative diseases such as Alzheimer’s disease and Parkinson’s disease, which were not included in the survey items of KNHANES [48,51]. Thus, such variables could not be considered in the analysis. Third, recall bias as well as individuals’ perceptual differences can result in errors in participants’ responses. For instance, one of the questions on KNHANES was “Did you usually accompany someone else when you had lunch over the last year?” which is undermined by its dependence on the flawed memories of participants. Meanwhile, internal standards that people possess regarding “how they usually” dined with companions could vary. Finally, the case of voluntary meals alone, which is known to offer an opportunity for refreshment, could not be distinguished from that of involuntary meals alone by the data preprocessing procedure [37]. This ignores the notion that dining alone is not always positively associated with depression. These drawbacks can be overcome by attempting case-control studies or by using different questionnaire formats, whose comparisons with this study could provide meaningful insights.

In conclusion, this study uncovered the relationship between dining with companions and the prevalence of depression. People who have meals with companions at least once a day tend to be less depressive than their counterparts who dine alone. More findings on companionship-related meal patterns and their concrete mechanisms of developing depression may be sought in future research.

## Figures and Tables

**Table 1:** Socioeconomic and health-related characteristics of the participants according to the presence of depression.

Variables	Male						Female					
	Total	Depression (PHQ ≥ 10)		No Depression (PHQ < 10)		*p*-Value	Total	Depressive (PHQ ≥ 10)		No Depression (PHQ < 10)		*p*-Value
		N	(%)	N	(%)			N	(%)	N	(%)	
**Meals together**						<0.0001						<0.0001
None	426	46	(10.8)	380	(89.2)		818	98	(12.0)	720	(88.0)	
Once or more	4273	87	(2.0)	4186	(98.0)		5538	236	(4.3)	5302	(95.7)	
**Age**						0.3764						<0.0001
19–29	462	10	(2.2)	452	(97.8)		463	27	(5.8)	436	(94.2)	
30–39	521	20	(3.8)	501	(96.2)		868	39	(4.5)	829	(95.5)	
40–49	758	18	(2.4)	740	(97.6)		1169	32	(2.7)	1137	(97.3)	
50–59	898	22	(2.4)	876	(97.6)		1350	67	(5.0)	1283	(95.0)	
60–69	1052	28	(2.7)	1024	(97.3)		1365	86	(6.3)	1279	(93.7)	
≥70	1008	35	(3.5)	973	(96.5)		1141	83	(7.3)	1058	(92.7)	
**Educational attainment**						0.0009						<0.0001
Middle school or below	1101	48	(4.4)	1053	(95.6)		2275	182	(8.0)	2093	(92.0)	
High school	1295	37	(2.9)	1258	(97.1)		1668	82	(4.9)	1586	(95.1)	
University or above	2303	48	(2.1)	2255	(97.9)		2413	70	(2.9)	2343	(97.1)	
**Equalized household income**						<0.0001						<0.0001
Quartile 1 (low)	810	61	(7.5)	749	(92.5)		1214	127	(10.5)	1087	(89.5)	
Quartile 2	1089	29	(2.7)	1060	(97.3)		1616	98	(6.1)	1518	(93.9)	
Quartile 3	1301	23	(1.8)	1278	(98.2)		1700	74	(4.4)	1626	(95.6)	
Quartile 4 (high)	1499	20	(1.3)	1479	(98.7)		1826	35	(1.9)	1791	(98.1)	
**Marital status**						<0.0001						<0.0001
Married	3653	82	(2.2)	3571	(97.8)		4601	189	(4.1)	4412	(95.9)	
Separated/divorced/widowed	299	21	(7.0)	278	(93.0)		1158	109	(9.4)	1049	(90.6)	
Never married	747	30	(4.0)	717	(96.0)		597	36	(6.0)	561	(94.0)	
**Smoking status**						0.0112						<0.0001
Non-smoker	1184	21	(1.8)	1163	(98.2)		5920	280	(4.7)	5640	(95.3)	
Smoker	3515	112	(3.2)	3403	(96.8)		436	54	(12.4)	382	(87.6)	
**Alcohol use status**						0.8974						0.2725
No	236	7	(3.0)	229	(97.0)		1151	68	(5.9)	1083	(94.1)	
Yes	4463	126	(2.8)	4337	(97.2)		5205	266	(5.1)	4939	(94.9)	
**Residential area**						0.7469						0.4644
Urban	2126	62	(2.9)	2064	(97.1)		2959	149	(5.0)	2810	(95.0)	
Rural	2573	71	(2.8)	2502	(97.2)		3397	185	(5.4)	3212	(94.6)	
**BMI**						0.0038						0.1007
Underweight	102	8	(7.8)	94	(92.2)		268	16	(6.0)	252	(94.0)	
Normal weight	1456	52	(3.6)	1404	(96.4)		2829	141	(5.0)	2688	(95.0)	
Overweight	1269	27	(2.1)	1242	(97.9)		1377	72	(5.2)	1305	(94.8)	
Obesity	1649	41	(2.5)	1608	(97.5)		1595	80	(5.0)	1515	(95.0)	
Severe obesity	223	5	(2.2)	218	(97.8)		287	25	(8.7)	262	(91.3)	
**Number of household members**						<0.0001						<0.0001
1	421	30	(7.1)	391	(92.9)		762	73	(9.6)	689	(90.4)	
2	1635	49	(3.0)	1586	(97.0)		1986	114	(5.7)	1872	(94.3)	
More than 2	2643	54	(2.0)	2589	(98.0)		3608	147	(4.1)	3461	(95.9)	
**Physical activity**						0.2715						0.7956
Low	1812	56	(3.1)	1756	(96.9)		2846	155	(5.4)	2691	(94.6)	
Moderate	2041	60	(2.9)	1981	(97.1)		2852	144	(5.0)	2708	(95.0)	
High	846	17	(2.0)	829	(98.0)		658	35	(5.3)	623	(94.7)	
**Year**						0.6128						0.0005
2014	1170	38	(3.2)	1132	(96.8)		1650	115	(7.0)	1535	(93.0)	
2016	1253	38	(3.0)	1215	(97.0)		1737	94	(5.4)	1643	(94.6)	
2018	1310	33	(2.5)	1277	(97.5)		1740	66	(3.8)	1674	(96.2)	
2020	966	24	(2.5)	942	(97.5)		1229	59	(4.8)	1170	(95.2)	
**Participants**	4699	133	(2.8)	4566	(97.2)		6356	334	(5.3)	6022	(94.7)	

Variables are presented with numbers and percentages: PHQ-9: Patient Health Questionnaire-9; BMI: Body Mass Index; Underweight: BMI < 18.5; Normal weight: 18.5 ≤ BMI < 23; Overweight: 23 ≤ BMI < 25; Obesity: 25 ≤ BMI < 30; Severe obesity: 30 ≤ BMI.

**Table 2:** Multivariate logistic regression analysis for the association between having meals together, socioeconomic/health-related variables, and depression.

Variables	Male		Female	
	Depression (PHQ ≥ 10)		Depression (PHQ ≥ 10)	
	Adjusted OR	95% CI	Adjusted OR	95% CI
**Meals together**				
None	1.00		1.00	
Once or more	0.26	(0.15–0.45)	0.50	(0.34–0.74)
**Age**				
19–29	1.00		1.00	
30–39	3.03	(1.23–7.48)	0.69	(0.27–1.78)
40–49	1.37	(0.53–3.58)	0.44	(0.17–1.12)
50–59	1.31	(0.48–3.56)	0.64	(0.25–1.62)
60-69	0.72	(0.27–1.93)	0.48	(0.18–1.29)
≥70	0.61	(0.21–1.76)	0.33	(0.12–0.90)
**Educational attainment**				
Middle school or below	1.00		1.00	
High school	1.04	(0.61–1.76)	0.64	(0.42–0.98)
University or above	0.71	(0.40–1.26)	0.33	(0.19–0.56)
**Equalized household income**				
Quartile 1 (low)	1.00		1.00	
Quartile 2	0.27	(0.15–0.49)	0.71	(0.48–1.04)
Quartile 3	0.21	(0.11–0.41)	0.51	(0.33–0.79)
Quartile 4 (high)	0.21	(0.10–0.43)	0.28	(0.17–0.48)
**Marital status**				
Married	1.00		1.00	
Separated/divorced/widowed	1.25	(0.56–2.80)	1.47	(1.03–2.09)
Never married	1.44	(0.73–2.81)	1.50	(0.69–3.31)
**Smoking status**				
Non-smoker	1.00		1.00	
Smoker	2.07	(1.11–3.87)	2.77	(1.90–4.06)
**Alcohol use status**				
No	1.00		1.00	
Yes	0.88	(0.32–2.46)	0.95	(0.68–1.34)
**Residential area**				
Urban	1.00		1.00	
Rural	0.71	(0.47–1.09)	1.06	(0.79–1.42)
**BMI**				
Underweight	1.97	(0.61–6.32)	1.32	(0.71–2.43)
Normal weight	1.00		1.00	
Overweight	0.56	(0.31–1.01)	0.95	(0.67–1.35)
Obesity	0.81	(0.48–1.37)	0.88	(0.61–1.26)
Severe obesity	0.58	(0.23–1.49)	1.36	(0.76–2.46)
**Number of family members**				
1	1.00		1.00	
2	0.91	(0.42–1.95)	1.42	(0.91–2.22)
More than 2	1.00	(0.47–2.13)	1.56	(0.96–2.54)
**Physical activity**				
Low	1.00		1.00	
Moderate	0.80	(0.43–1.50)	1.07	(0.66–1.72)
High	0.99	(0.62–1.57)	0.93	(0.69–1.25)
**Year**				
2014	1.85	(1.05–3.25)	1.84	(1.21–2.80)
2016	1.44	(0.78–2.65)	1.40	(0.93–2.11)
2018	1.00		1.00	
2020	1.56	(0.79–3.08)	1.33	(0.84–2.10)

PHQ-9: Patient Health Questionnaire-9; BMI: Body Mass Index; Underweight: BMI < 18.5; Normal weight: 18.5 ≤ BMI < 23; Overweight: 23 ≤ BMI < 25; Obesity: 25 ≤ BMI < 30; Severe obesity: 30 ≤ BMI.

**Table 3:** Subgroup analysis of the association between having meals together and depression stratified by demographic variables.

Variables	Male			Female		
	Meals Alone	Meals Together		Meals Alone	Meals Together	
	Adjusted OR	Adjusted OR	95% CI	Adjusted OR	Adjusted OR	95% CI
**Age**						
19–29	1.00	0.07	(0.01–0.37)	1.00	0.12	(0.02–0.70)
30–39	1.00	0.41	(0.06–2.68)	1.00	0.52	(0.13–2.17)
40–49	1.00	0.42	(0.08–2.11)	1.00	0.26	(0.08–0.89)
50–59	1.00	0.29	(0.10–0.87)	1.00	0.69	(0.28–1.69)
60–69	1.00	0.10	(0.03–0.35)	1.00	0.45	(0.23–0.87)
≥70	1.00	0.18	(0.08–0.44)	1.00	0.49	(0.25–0.97)
**Educational attainment**						
Middle school or below	1.00	0.16	(0.07–0.39)	1.00	0.51	(0.32–0.81)
High school	1.00	0.34	(0.12–0.96)	1.00	0.60	(0.23–1.59)
University or above	1.00	0.21	(0.08–0.54)	1.00	0.40	(0.15–1.08)
**Equalized household income**						
Quartile 1 (low)	1.00	0.16	(0.06–0.47)	1.00	0.58	(0.30–1.09)
Quartile 2	1.00	0.41	(0.14–1.23)	1.00	0.40	(0.21–0.79)
Quartile 3	1.00	0.13	(0.03–0.53)	1.00	0.30	(0.13–0.68)
Quartile 4 (high)	1.00	0.27	(0.08–0.94)	1.00	0.42	(0.08–2.15)
**Marital status**						
Married	1.00	0.37	(0.16–0.83)	1.00	0.61	(0.34–1.10)
Separated/divorced/widowed	1.00	0.02	(0.00–0.30)	1.00	0.44	(0.25–0.79)
Never married	1.00	0.20	(0.07–0.51)	1.00	0.26	(0.06–1.13)
**Smoking status**						
Non-smoker	1.00	0.15	(0.05–0.46)	1.00	0.45	(0.29–0.69)
Smoker	1.00	0.30	(0.16–0.56)	1.00	0.89	(0.35–2.26)
**Alcohol use status**						
No	1.00	0.08	(<0.001–>999.999)	1.00	0.54	(0.25–1.18)
Yes	1.00	0.24	(0.13–0.43)	1.00	0.50	(0.32–0.79)
**Residential area**						
Urban	1.00	0.17	(0.07–0.41)	1.00	0.47	(0.25–0.86)
Rural	1.00	0.33	(0.16–0.71)	1.00	0.57	(0.33–0.97)
**BMI**						
Underweight	1.00	220.86	(0.98–>999.999)	1.00	0.10	(0.01–0.91)
Normal weight	1.00	0.12	(0.05–0.30)	1.00	0.40	(0.22–0.74)
Overweight	1.00	0.16	(0.05–0.52)	1.00	0.90	(0.45–1.81)
Obesity	1.00	0.37	(0.12–1.14)	1.00	0.37	(0.17–0.83)
Severe obesity	1.00	0.07	(0.01–0.73)	1.00	0.77	(0.23–2.57)
**Number of household members**						
1	1.00	0.13	(0.05–0.39)	1.00	0.34	(0.15–0.79)
2	1.00	0.42	(0.14–1.30)	1.00	0.59	(0.32–1.10)
More than 2	1.00	0.19	(0.08–0.43)	1.00	0.48	(0.24–0.97)
**Physical activity**						
Low	1.00	0.04	(0.01–0.23)	1.00	1.60	(0.37–6.89)
Moderate	1.00	0.55	(0.22–1.38)	1.00	0.33	(0.18–0.62)
High	1.00	0.14	(0.05–0.38)	1.00	0.65	(0.38–1.13)
**Year**						
2014	1.00	0.52	(0.14–1.90)	1.00	0.43	(0.22–0.86)
2016	1.00	0.05	(0.02–0.16)	1.00	0.68	(0.29–1.64)
2018	1.00	0.77	(0.21–2.86)	1.00	0.29	(0.14–0.61)
2020	1.00	0.14	(0.04–0.48)	1.00	0.80	(0.38–1.67)

PHQ-9: Patient Health Questionnaire-9; BMI: Body Mass Index; Underweight: BMI < 18.5; Normal weight: 18.5 ≤ BMI < 23; Overweight: 23 ≤ BMI < 25; Obesity: 25 ≤ BMI < 30; Severe obesity: 30 ≤ BMI.

**Table 4:** Subgroup analysis of the association between having meals together and depression stratified by types of meal and types of companions with whom participants had meals.

Meals Together		Male		Female	
		Depression (PHQ ≥ 10)		Depression (PHQ ≥ 10)	
		Adjusted OR	95% CI	Adjusted OR	95% CI
**Breakfast together**					
No		1.00		1.00	
Yes					
	With family	0.87	(0.52–1.44)	0.91	(0.67–1.23)
	With people other than family	1.12	(0.39–3.16)	0.50	(0.16–1.61)
**Lunch together**					
No		1.00		1.00	
Yes					
	With family	0.66	(0.38–1.13)	1.07	(0.75–1.53)
	With people other than family	0.33	(0.20–0.55)	0.63	(0.42–0.94)
**Dinner together**					
No		1.00		1.00	
Yes					
	With family	0.46	(0.27–0.79)	0.63	(0.43–0.90)
	With people other than family	0.25	(0.09–0.68)	0.70	(0.37–1.33)

PHQ-9: Patient Health Questionnaire-9.

## Data Availability

Data used in this study were from 2014, 2016, 2018, 2020 KNHANES. Raw data as a whole is available to the public, and data can be downloaded from the KNHANES official website (http://knhanes.kdca.go.kr/, accessed on 23 March 2022).

## Supplementary Materials

The following supporting information can be downloaded at: https://www.mdpi.com/article/10.3390/nu14142834/s1, Table S1: Numbers and percentages of participants who dined alone or together according to the detailed levels of depression; Table S2: Numbers and percentages of participants who dined alone or together according to types of meals and types of companions; Table S3: Subgroup analysis of the association between having meals together and depression stratified by several meal-pattern variables.
