# Supplementary material for: Association between Dining with Companions and Depression among Korean Adults
Source: Nutrients. 2022 Jul 10;14(14):2834. doi: 10.3390/nu14142834 (PMC9325312; doi:10.3390/nu14142834)
Supplement: Supplementary file 1 [file nutrients-14-02834-s001.zip › nutrients-1783361-supplementary.pdf]

Table S1. Numbers and percentages of participants who dined alone or together according to the detailed levels of depression.

Meals together	No depression (PHQ<5)		Mild depression (5≤PHQ<10)		Moderate depression (10≤PHQ<20)		Severe depression (PHQ≥20)		p-value
	N	(%)	N	(%)	N	(%)	N	(%)	
**Male**									<0.0001
None	322	(75.6)	58	(13.6)	41	(9.6)	5	(1.2)	
Once or more	3827	(89.6)	359	(8.4)	80	(1.9)	7	(0.2)	
**Total**	4149		417		121		12		
**Female**									<0.0001
None	591	(72.2)	129	(15.8)	85	(10.4)	13	(1.6)	
Once or more	4515	(81.5)	787	(14.2)	223	(4.0)	13	(0.2)	
**Total**	5106		916		308		26		

PHQ-9: Patient Health Questionnaire-9

Table S2. Numbers and percentages of participants who dined alone or together according to types of meals and types of companions.

Meals together	Male						Female					
	Total	Depression (PHQ≥10)		No Depression (PHQ<10)		p-value	Total	Depression (PHQ≥10)		No Depression (PHQ<10)		p-value
		N	(%)	N	(%)			N	(%)	N	(%)	
**Breakfast together**						0.0159						0.0002
No	1806	67	3.7	1739	96.3		2617	173	6.6	2444	93.4	
Yes												
With family	2653	61	2.3	2592	97.7		3619	157	4.3	3462	95.7	
With people other than family	240	5	2.1	235	97.9		120	4	3.3	116	96.7	
**Lunch together**						<0.0001						<0.0001
No	1196	62	5.2	1134	94.8		2659	180	6.8	2479	93.2	
Yes												
With family	1219	38	3.1	1181	96.9		1606	92	5.7	1514	94.3	
With people other than family	2284	33	1.4	2251	98.6		2091	62	3.0	2029	97.0	
**Dinner together**						<0.0001						<0.0001
No	884	56	6.3	828	93.7		1526	126	8.3	1400	91.7	
Yes												
With family	3196	71	2.2	3125	97.8		4474	189	4.2	4285	95.8	
With people other than family	619	6	1.0	613	99.0		356	19	5.3	337	94.7	

PHQ-9: Patient Health Questionnaire-9

Table S3. Subgroup analysis of the association between having meals together and depression stratified by several meal-pattern variables.

Meals together	Male		Female	
	Depression (PHQ≥10)		Depression (PHQ≥10)	
	Adjusted OR	95% CI	Adjusted OR	95% CI
**Frequency of breakfast per week**				
None				
3 ~ 4 times	1.00		1.00	
5 ~ 7 times	1.44	(0.48 - 4.38)	1.25	(0.44 - 3.58)
Once or more				
3 ~ 4 times	0.34	(0.10 - 1.11)	0.82	(0.28 - 2.43)
5 ~ 7 times	0.35	(0.11 - 1.08)	0.57	(0.20 - 1.62)
**Frequency of lunch per week**				
None				
3 ~ 4 times	1.00		1.00	
5 ~ 7 times	2.48	(0.71 - 8.68)	0.82	(0.34 - 1.98)
Once or more				
3 ~ 4 times	1.30	(2.26 - 6.47)	0.43	(0.16 - 1.17)
5 ~ 7 times	0.56	(0.16 - 1.99)	0.42	(0.18 - 0.98)
**Frequency of dinner per week**				
None				
3 ~ 4 times	1.00		1.00	
5 ~ 7 times	0.41	(0.12 - 1.42)	0.45	(0.18 - 1.11)
Once or more				
3 ~ 4 times	0.22	(0.05 - 1.04)	0.26	(0.09 - 0.75)
5 ~ 7 times	0.11	(0.04 - 0.36)	0.25	(0.10 - 0.61)
**Whether one had breakfast yesterday**				
None				
No	1.00		1.00	
Yes	0.63	(0.20 - 2.00)	1.13	(0.31 - 4.08)
Once or more				
No	0.31	(0.09 - 1.03)	0.65	(0.16 - 2.60)
Yes	0.16	(0.05 - 0.47)	0.54	(0.15 - 1.99)
**Whether one had lunch yesterday**				
None				
No	1.00		1.00	
Yes	3.09	(0.46 - 20.59)	1.53	(0.55 - 4.26)
Once or more				
No	1.53	(0.21 - 11.34)	0.90	(0.26 - 3.13)
Yes	0.70	(0.11 - 4.68)	0.73	(0.26 - 2.07)

**Whether one had dinner yesterday**									
None									
No	1.00				1.00				
Yes	2.06	(	0.25	-	17.09)	0.84	(0.32	-	2.25)
Once or more									
No	1.37	(	0.13	-	14.27)	0.70	(0.24	-	2.06)
Yes	0.49	(	0.06	-	3.99)	0.41	(0.16	-	1.07)
**Number of meals yesterday**									
None									
1	1.00					1.00			
2	1.12	(	0.11	-	11.32)	1.37	(0.14	-	13.01)
3	1.24	(	0.13	-	12.20)	1.50	(0.17	-	12.91)
Once or more									
0	No data					17.14	(1.06	-	277.43)
1	1.31	(	0.09	-	20.17)	1.01	(0.07	-	15.13)
2	0.52	(	0.06	-	4.75)	0.85	(0.10	-	7.23)
3	0.25	(	0.03	-	2.23)	0.60	(0.08	-	5.82)

Meal-patterns specified "yesterday" are referred to as data of the day before the corresponding investigation was performed. PHQ-9: Patient Health Questionnaire-9
